# P01.33. A new development of Triterpene Acids-containing extracts from Viscum album L. displays synergistic induction of apoptosis in childhood leukemia

**DOI:** 10.1186/1472-6882-12-S1-P33

**Published:** 2012-06-12

**Authors:** C Delebinski, S Jäger, G Kauczor, K Kemnitz-Hassanin, K Seeger, G Henze, H Lode, G Seifert

**Affiliations:** 1 Charité, Department of Pediatric Oncology/Hematology, Berlin, Germany; 2 Betulin-Institute , Darmstadt, Germany; 3 Department of Paediatric Haematology and Oncology, Greifswald, Germany

## Purpose

Aqueous Viscum album L. extracts (VAE) are widely used in complementary cancer therapies. Due to their low solubility, triterpene acids, which are known to possess anti-cancer properties, do not occur in aqueous extracts in significant amounts. Using cyclodextrins it was possible to solubilize mistletoe triterpene acids and to determine the anti-cancer properties in different acute lymphoblastic (ALL) and myeloid leukemia cell lines (AML).

## Methods

The experimental extracts contain either mistletoe lectin-I and viscotoxins (viscum) or solubilized oleanolic- and betulinic acids (TT) and more interestingly, a combination thereof (viscumTT). The cytotoxicity of increasing concentrations of VAE preparations was tested in NALM-6, U937 and HL-60 cells *in vitro*. Apoptosis was determined using mitochondrial membrane potential measurement, Annexin/PI, Western blot analysis and caspase assays. A C.B-17/SCID model of pre-B ALL/NALM-6 was used to test efficacy and mechanisms of treatment with lectin- and triterpene-containing preparations *in vivo*.

## Results

All three cell lines have shown distinct apoptosis induction for viscum, TT and viscumTT. However, differences between ALL and AML cell lines toward the lectin and triterpene acids sensitivity were observed. Annexin/PI and mitochondrial membrane potential assays indicated that dose-dependent induction of apoptosis was the main mechanism. The combination (viscumTT) of lectin- (viscum) and triterpene acids-containing (TT) extracts showed the strongest apoptosis induction. Furthermore, caspase activity demonstrated that these extracts are able to induce apoptosis via caspase-8 and -9 dependent pathways. The *in vivo* experiment showed that treatment of mice with the viscumTT combination prolonged the mean survival significantly compared control group.

## Conclusion

Taken together, we were able to show that this new formulation “viscumTT” of aqueous mistletoe extracts and triterpene acids can induce apoptosis in leukemia cells via the intrinsic and extrinsic signaling pathways. Based on these data we believe that Viscum album L. extracts containing triterpene acids may possess impressive therapeutic potential.

